# Small-molecule survivin inhibitor YM155 enhances radiosensitization in esophageal squamous cell carcinoma by the abrogation of G_2_ checkpoint and suppression of homologous recombination repair

**DOI:** 10.1186/s13045-014-0062-8

**Published:** 2014-08-20

**Authors:** Qin Qin, Hongyan Cheng, Jing Lu, Liangliang Zhan, Jianchao Zheng, Jing Cai, Xi Yang, Liping Xu, Hongcheng Zhu, Chi Zhang, Jia Liu, Jianxin Ma, Xizhi Zhang, Shengbin Dai, Xinchen Sun

**Affiliations:** 1 Department of Radiation Oncology, the First Affiliated Hospital of Nanjing Medical University, No.300 Guangzhou Road, Nanjing 210029, China; 2 Department of Physiology, Nanjing Medical University, Nanjing, China; 3 Department of Radiation Oncology, Nantong Tumor Hospital, Nantong, China; 4 Department of Radiation Oncology, The No.2 People’s Hospital of Lian Yungang, Lianyungang, China; 5 Department of Radiation Oncology, Subei People’s Hospital, Yangzhou, China; 6 Department of Radiation Oncology, People’s Hospital of Tai Zhou, Taizhou, China

**Keywords:** Survivin, YM155, Radiosensitization, ESCC, G2 checkpoint, Homologous recombination repair

## Abstract

**Background:**

Survivin is overexpressed in cancer cells and plays a crucial role in apoptosis evasion. YM155, a small-molecule inhibitor of survivin, could enhance the cytotoxicity of various DNA-damaging agents. Here, we evaluated the radiosensitizaion potential of YM155 in human esophageal squamous cell carcinoma (ESCC).

**Methods:**

Cell viability was determined by CCK8 assay. The radiosensitization effect of YM155 was evaluated by clonogenic survival and progression of tumor xenograft. Cell cycle progression was determined by flow cytometric analysis. Radiation-induced DNA double strand break (DSB) and homologous recombination repair (HRR) were detected by the staining of γ-H2AX and RAD51, respectively. Expression of survivin and cell cycle regulators was detected by Western blot analysis.

**Results:**

YM155 induced radiosensitization in ESCC cell lines Eca109 and TE13, associated with the abrogation of radiation induced G_2_/M checkpoint, impaired Rad51 focus formation, and the prolongation of γ-H2AX signaling. G_2_/M transition markers, including the activation of cyclinB1/Cdc2 kinase and the suppression of Cdc2 Thr14/Tyr15 phosphorylation were induced by YM155 in irradiated cells. The combination of YM155 plus irradiation delayed the growth of ESCC tumor xenografts to a greater extent compared with either treatment modality alone.

**Conclusions:**

Our findings suggest that the abrogation of G_2_ checkpoint and the inhibition of HRR contribute to radiosensitization by YM155 in ESCC cells.

## Background

Survivin is the smallest member of the inhibitor of apoptosis protein (IAP) family with a molecular mass of 16.5 kD. Survivin functions to inhibit mitochondrial pathway of apoptosis and regulate cell division as a component of the spindle checkpoint machinery [1]. Overexpression of survivin was detected in many types of cancers including esophageal squamous cell carcinoma (ESCC) but rarely in normal differentiated adult tissues [2]–[4]. Furthermore, elevated levels of survivin have been associated with poor prognosis in several human cancers [5]–[9]. In ESCC patients high levels of survivin expression indicated poor prognosis as well as high resistance to radiotherapy and chemotherapy [10],[11]. Accordingly, the suppression of survivin expression with the use of antisense oligonucleotides or ribozymes effectively overcame apoptosis resistance in different types of cancer cells and sensitized cancer cells to radiation and chemotherapeutic agents in vitro and in vivo [12]–[16].

It is thought that survivin enhances tumor cell survival primarily through the suppression of apoptosis-related cell death via direct inhibition of caspase-related proteins. However, it has become increasingly clear that the role of survivin in response to ionizing radiation far exceeds a simple inhibition of apoptotic pathway. The underlying molecular mechanisms seem to be multifaceted and involve broader cellular adaptation processes within separate subcellular compartments. Chakravarti *et al.* are the first to report on novel caspase-independent mechanisms through which survivin enhances tumor cell survival upon radiation exposure, including the regulation of double-strand DNA break repair and cell metabolism [12]. Recently, Reichert *et al.* found a direct relationship between survivin and components of the DNA-double strand break (DSB) repair machinery following irradiation in radiation resistant glioblastoma cells [17].

In the nucleus, survivin is selectively expressed at G_2_/M phase of the cell cycle and localized to microtubules of the mitotic spindle, thus performing the role of the regulator of cell division [18]. Connor *et al.* showed that survivin underwent cell cycle-dependent phosphorylation on Thr34 by a Cdc2/cyclin B1 complex, which was required to prevent from caspase-9-dependent apoptosis of cells traversing mitosis and preserve cell viability at cell division [19]. Thus we speculated that the attenuation of survivin expression is expected to impact DNA damage induced G_2_/M checkpoint.

YM155 was identified as a first-in-class small molecule inhibitor of survivin. YM155 selectively inhibited survivin expression at both mRNA and protein levels at subnanomolar range and exhibited anticancer activity in preclinical models of several types of cancers [20]–[22]. However, the effectiveness of YM155 with ESCC has not been confirmed. In the present study, we employed two ESCC cell lines Eca109 and TE-13 to evaluate the radiosensitizing effects of YM155 on ESCC, with a special emphasis on its interference with cell cycle checkpoint.

## Results

### YM155 selectively reduced the expression of survivin in ESCC cells

**Figure 1:**
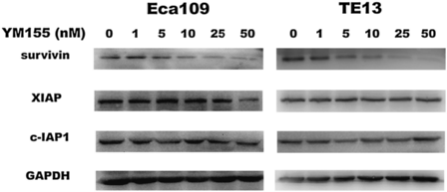
**YM155 suppresses survivin expression in a dose dependent manner in human ESCC cells.** Eca109 and TE13 cells were treated with 1, 5, 10, 25, or 50 nmol/L YM155, or DMSO as control for 48 h. Protein expression levels of IAP family members were determined by Western blot analysis. GAPDH was used as loading control.

First, we assessed the effect of YM155 on survivin expression in two ESCC cell lines Eca109 and TE13. Western blot analysis showed that YM155 inhibited survivin expression in a dose dependent manner, but had no significant effect on the abundance of other IAP family members such as XIAP and c-IAP1 (Figure 1). These results suggest that YM155 specifically suppresses survivin at low nanomolar concentrations in ESCC cells.

### YM155 enhanced cytotoxicity of irradiation in ESCC cells

**Figure 2:**
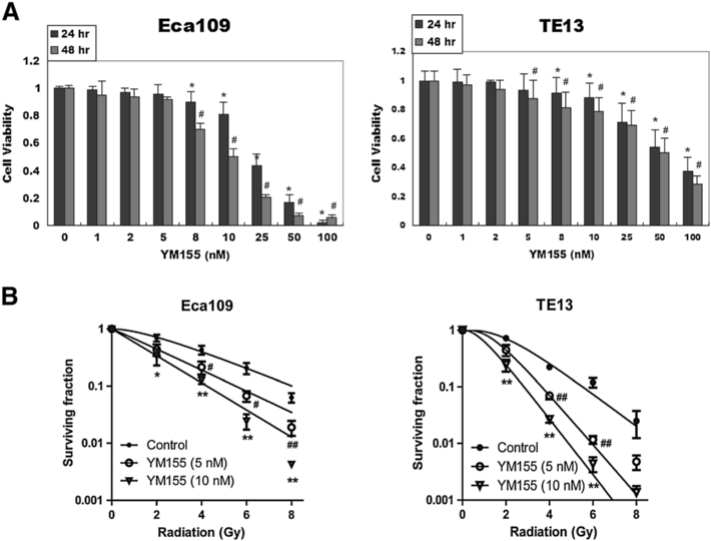
**YM155 sensitizes ESCC cells to irradiation. A**, ESCC cell lines Eca109 and TE13 were seeded in 96-well plates in triplicate and treated with various concentrations of YM155 for 24 or 48 h. Cell viability was determined by CCK8 assay. * and #, *P* < 0.05 versus control. **B**, Cell survival curve was established by clonogenic survival assay. The cells were treated with 5 nM or 10 nM YM155 and ionizing radiation as illustrated and harvested after incubation for 10–14 d. Each bar represented mean ± SE from 3 independent experiments. * and #, *P* < 0.05 versus control; ** and ##, *P* < 0.01 versus control.

Next, we evaluated the viability of ESCC cells after 24- and 48- h incubation with increasing concentration of YM155. At 24 h, the IC50 of YM155 on Eca109 and TE13 cells were 21 and 60 nM, respectively. At 48 h, the IC50 of YM155 on Eca109 and TE13 cell lines were 12 and 50 nM, respectively (Figure 2A). The sub-toxic concentrations of YM155 (5 nM and 10 nM) were adopted to investigate the radiosensitivity of the two cell lines.

Colony-forming assay with ESCC cells showed that YM155 promoted radiation-induced clonogenic cell death in a dose dependent manner. When the concentration of YM155 reached 10 nM, the SER (sensitization enhancement ratio) of Eca109 and TE13 cells was 1.51 and 1.73, respectively. Radiobiological variables were calculated and summarized in Table 1. These data indicate that YM155 remarkably enhanced cell death in irradiated ESCC cells.

**Table 1:** Radiosensitization effects of YM155 on ESCC cells in vitro

	**D**_**0**_**(Mean inactivation dose)**	**Dq**	**Surviving fraction (2 Gy)**	**Sensitization enhancement ratio**
**Eca109**				
Control	2.74	1.78	0.72	
YM155 (5 nM)	2.34	0.13	0.44	1.20
YM155 (10 nM)	1.82	0.03	0.34	1.51
**TE13**				
Control	1.52	1.97	0.71	
YM155 (5 nM)	0.98	1.41	0.44	1.55
YM155 (10 nM)	0.88	0.82	0.24	1.73

### YM155 reduced irradiation induced accumulation of G_2_/M fraction in ESCC cells

**Figure 3:**
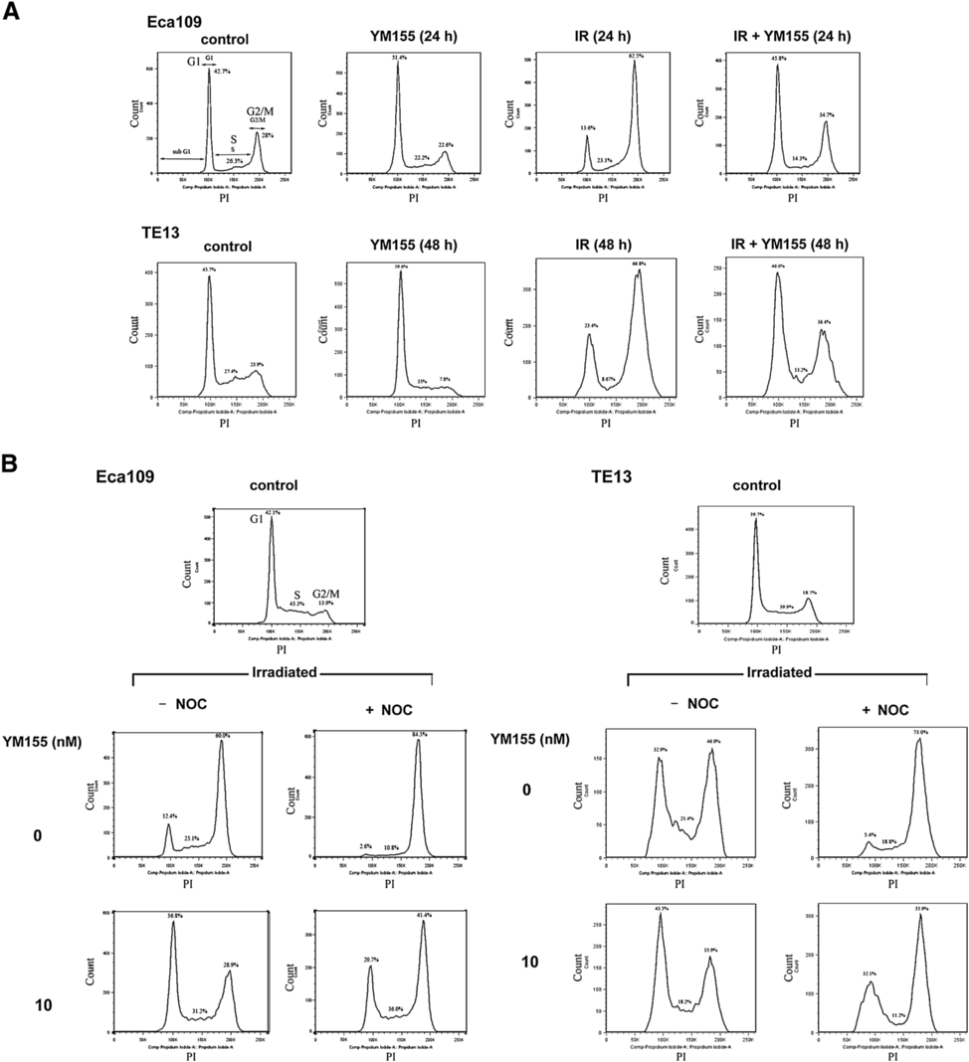
**YM155 abrogates radiation-induced G**_**2**_**arrest in ESCC cells. A.** Eca109 and TE13 cells were treated with YM155, X ray or the combination modality and then harvested for analysis of cell cycle distribution by flow cytometry. **B.** Cells treated with X ray alone or in combination with YM155 were then incubated in the absence (left panels) or the presence (right panels) of nocodazole (NOC, 0.4 μg/mL) for 24 h. Shown were representative flow cytometry plots of three experiments with similar results.

To explore the effect of survivin inhibitor on radiation-induced cell cycle checkpoint, we performed cytometric analysis on ESCC cells exposed to 8 Gy of X rays. The results showed that both two cell lines were arrested in G_2_ phase of cell cycle (62.5% for Eca109 and 66.1% for TE13). Radiation-induced G_2_/M arrest was abrogated by 10 nM YM155 (34.7% for Eca109 and 36.4% for TE13), with a concomitant rise in G_1_ and S phases (Figure 3A and B). Exposure of the cells to YM155 alone caused small decrease in G_2_/M fraction and slight accumulation of G_1_ population (Figure 3A). In order to confirm that YM155 abrogated G_2_ arrest, rather than induced a G_1_/S- phase block, mitotic inhibitor nocodazole was used. As shown in Figure 3B, the addition of nocodazole (0.4 μg/mL) successfully prevented irradiated cells exposed to YM155 from cell cycle progression. Therefore, YM155 seems to impact the progression of cells from G_2_ to M phase. Detailed data of cell cycle distribution were summarized in Table 2.

**Table 2:** Abrogation of G2/M checkpoint by YM155 in ESCC cells

	**Eca109**			**TE13**		
**Treatment**	**G**_**1**_**(%)**	**S (%)**	**G**_**2**_**/M (%)**	**G**_**1**_**(%)**	**S (%)**	**G**_**2**_**/M (%)**
**Control**	42.7	26.3	28.0	45.7	27.4	23.9
**YM155**	51.4	22.2	22.6	59.6	35	7.8
**IR**	13.6	23.1	62.5	23.4	8.7	66.8
**IR + YM155**	45.8	14.3	34.7	46.6	13.2	36.4
**Treatment**	**G**_**1**_**(%)**	**S (%)**	**G**_**2**_**/M (%)**	**G**_**1**_**(%)**	**S (%)**	**G**_**2**_**/M (%)**
**Control**	42.1	43.3	13.9	39.7	39.9	18.7
**IR**	12.4	25.1	60.0	32.9	21.4	46.0
**IR + Noc**	2.6	10.8	84.5	5.4	18.8	73.0
**IR + YM155**	36.8	31.2	28.9	43.5	18.2	35.9
**IR + YM155 + Noc**	20.7	36.0	41.4	32.1	11.2	55.9

### YM155 enhanced Cdc2 kinase activation and attenuated cyclin B1 expression in irradiated ESCC cells

**Figure 4:**
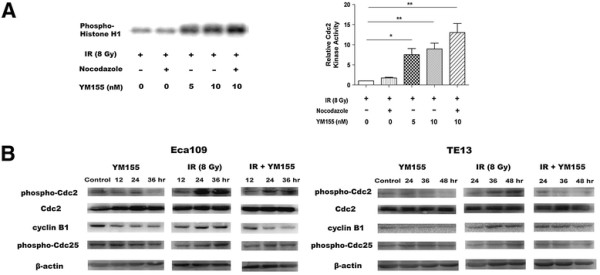
**YM155 potentiates the activation of Cdc2 kinase and suppresses protein expression related to G**_**2**_**/M checkpoint. A.** Irradiated Eca109 cells were treated with YM155 in the absence or the presence of nocodazole. Cdc2 was immunoprecipitated using Cdc2 antibody and Cdc2 kinase activity was measured by Histone H1 kinase assay described. The relative Cdc2 kinase activity was shown in the histogram (right) (*, *P* < 0.05; **, *P* < 0.01). **B.** Eca109 and TE13 cells were treated with YM155, X ray or the combination modality and subjected to Western blot analysis of G_2_/M checkpoint related proteins phospho-Cdc2, Cdc2, cyclin B1, phospho-Cdc25C.

Histone H1 kinase activity of Cdc2 was suppressed in irradiated Eca109 cells. However, YM155 resulted in significant activation of Cdc2 kinase in irradiated cells, and this effect was most evident when nocodazole was administered post-irradiation to prevent cells from existing M phase (Figure 4A). Cdc2/cyclin B1 complex is the key enzyme regulating G_2_ to M transition and is controlled by the phosphorylation at various sites. DNA damage induced G_2_ phase arrest is linked to the accumulation of relatively inactive, hyperphosphorylated Cdc2/cyclin B1 complexes which have inhibitory phosphates on two residues (Thr14/Tyr15) of Cdc2. To determine whether YM155 induced abrogation of G_2_ checkpoint is mediated by the suppression of Cdc2 inhibitory phosphorylation, we monitored the phosphorylation level of Cdc2 as well as cyclin B1 expression in Eca109 and TE13 cells. Western blot analysis showed that treatment with YM155 alone slightly suppressed basal level of phospho-Cdc2 and cyclin B1. In both ESCC cell lines, irradiation induced inhibitory phosphorylation of Cdc2 on T-14 and Y-15 and accumulation of cyclin B1, which was most obvious at 36 h for Eca109 and at 48 h for TE13. YM155 evidently abrogated X-ray induced Cdc2 hyperphosphorylation and cyclin B1 accumulation without affecting total Cdc2 and phospho-Cdc25C protein levels (Figure 4B).

### YM155 inhibited irradiation induced homologous recombination repair (HRR)

**Figure 5:**
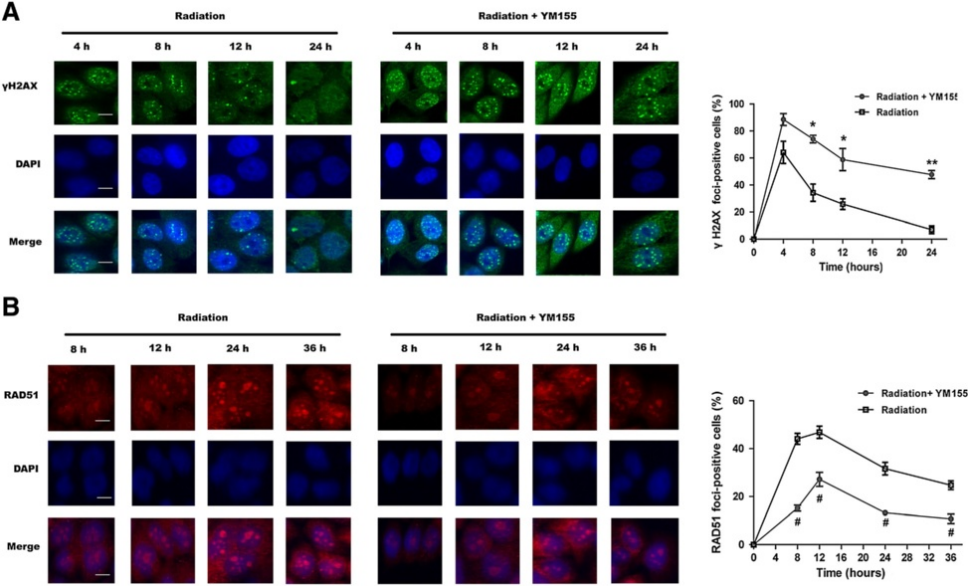
**YM155 suppresses radiation-induced DSB repair in ESCC cells. A.** YM155 led to significant increase in γ-H2AX staining. **B.** YM155 inhibited the formation of radiation-induced Rad51 foci. Eca109 cells were treated with 0.1% DMSO or 10 nM YM155 and then exposed to 4 Gy of X ray. After incubation for the indicated times, the cells were fixed for immunofluorescence detection of γ-H2AX (green) and Rad51 (red) foci. Representative images of nuclei (blue) and foci were shown (left lane), scale bar, 10 μm. Data were expressed as the percentage of cells staining positive for foci and plotted as the mean SEM of 3 independent experiments (right lane). Nuclei containing ≥10 immunoreactive foci were scored as positive for γ-H2AX, and ≥5 foci as positive for RAD51. At least 100 nuclei were counted for each experiment. * and #, *P* < 0.05; **, *P* < 0.01.

To further explore the mechanisms of YM155 induced abrogation of G_2_ arrest, we detected γ-H2AX and RAD51 foci in Eca109 cells. As anticipated, radiation produced a γ-H2AX signal as early as 30 min, which was reduced to basal level 24 h post irradiation (data not shown). However, YM155 treatment resulted in a significant prolongation of γ-H2AX signal at least 24 h post irradiation (47.7 ± 3.0%) compared with radiation alone (7 ± 2.8%; *P* < 0.001) (Figure 5A). These results suggest that YM155 significantly inhibited the repair of DSBs manifested as the persistence of γ-H2AX foci at 4 to 24 h after radiation. We next assessed RAD51 focus formation, as a hallmark of ongoing DNA homologous recombination repair (HRR). In response to radiation alone, Rad51 formed discrete nuclear foci at 8 h and the percentage of RAD51 foci-positive cells increased up to 36 h after treatment. However, in the presence of YM155, the assembly of Rad51 foci in response to radiation was significantly suppressed, which was in contrast with kinetics of γ-H2AX (Figure 5B). Taken together, these findings support the conclusion that YM155 inhibits HRR, likely through the inhibition of Rad51 focus formation in response to radiation.

### YM155 enhanced caspase-associated apoptosis in irradiated ESCC cells

**Figure 6:**
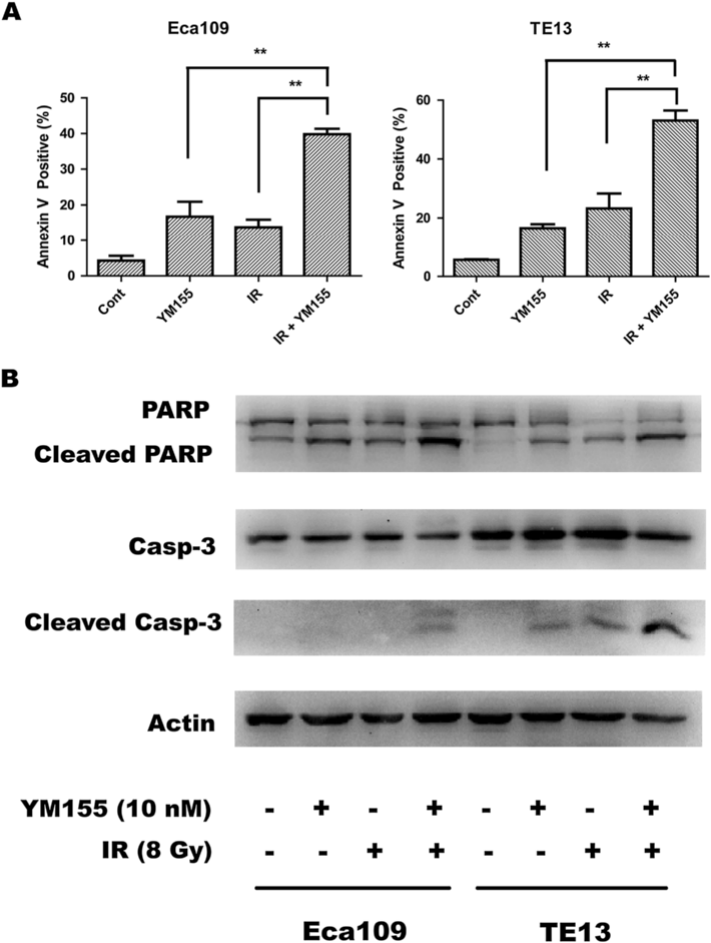
**YM155 enhances radiation induced apoptosis.** Cells were seeded into six-well plates at the concentration of 2 × 10^5^/mL, incubated with 10 nmol/L YM155 or 0.1% DMSO for 24 h, and (or not) irradiated at dose of 10 Gy, respectively. Forty-eight hours after incubation, cells were harvested. **A,** The cells were stained with AnnexinV/FITC and propidium iodide and examined by flow cytometry for apoptotic cell populations. Data were Mean ± SD (n = 3). **, *P* < 0.01. **B.** Western blot analysis of apoptosis-related proteins. Actin was used as a loading control.

To analyze the possible enhancement of radiation-induced apoptosis in ECSS cells after YM155 exposure, we first performed Annexin V/FITC and propidium iodide dual staining to quantify apoptotic cells after treatment with YM155 (10 nM) and radiation (8 Gy), alone and in combination for 48 h. As shown in Figure 6A, the induction of early apoptotic events (Annexin V positive) was most evident when cells were treated with 8 Gy plus YM155. The difference between groups of combinational and each single treatment was statistically significant (*P* < 0.01) in both Eca109 and TE13 cells. We next detected the cleavage of caspase-3 and PARP. As shown in Figure 6B, higher levels of cleaved PARP and caspase-3 were observed in Eca109 cells treated with 8 Gy radiation plus 10 nM YM155, compared with the cells treated with YM155 or radiation alone. Similar results were observed in TE13 cells.

### YM155 inhibited survivin expression and radiosensitized Eca109 in vivo xenograft tumor

**Figure 7:**
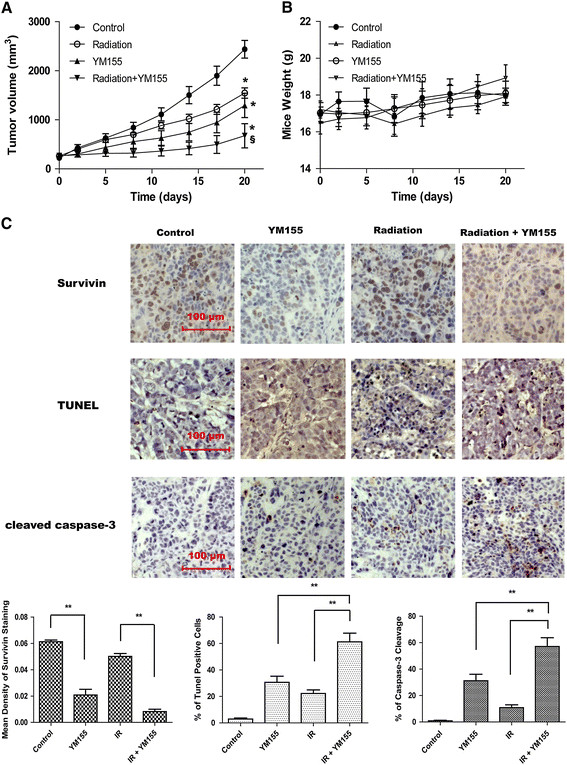
**YM155 inhibits Eca109 xenografts in response to radiation. A.** Tumor volume was measured at the indicated times after the onset of treatment. Data were Mean ± SD from six mice per group; *, *P* < 0.01, versus control; §, *P* < 0.05, YM155 plus IR group versus single treatment. **B.** Mice body weight of different treatments was measured at the indicated times. **C.** Immunohistochemistry analysis of survivin and cleaved caspase-3 expression and TUNEL analysis for apoptosis in tumor sections after different treatments. Histograms represented the mean intensity of survivin staining (left), the percent of apoptotic cells (middle), and the percent of positive staining of cleaved caspase-3 (right). **, *P* < 0.01.

To determine the in vivo radiosensitizing activity of YM155 for ESCC, mice bearing Eca109 cells xenograft tumor were utilized. As shown in Figure 7A, YM155 or irradiation alone produced significant tumor volume regression on day 20 (*P* = 0.006 for irradiation, *P* = 0.001 for YM155) compared to control group. However, the combination of YM155 and radiation produced more tumor volume regression (*P* < 0.05 for YM155 plus IR versus single treatment). Notably, the combined treatment significantly prolonged the time required for tumor volume doubling relative to radiation or YM155 alone (11.9 d vs. 2.3 or 3.3 d). No complete regression in either treatment alone was observed. The enhancement factor (EF) was 3.75. The tumor growth delay induced by each modality was summarized in Table 3. All kinds of the treatments were well tolerated by the animals with no evidence of local cutaneous damage or systemic toxicity such as weight loss (Figure 7B).

**Table 3:** Radiosensitization effects of YM155 on Eca109 xenograft mouse model

**Treatment**	**Doubling time (days)**	**Absolute growth delay (days)**^**a**^	**Normalized growth delay (days)**^**b**^	**Enhancement factor**
**Control**	5.7 ± 0.5	0		
**IR**	8.0 ± 2.0	2.3 (8.0-5.7)		
**YM155**	9.0 ± 1.6	3.3 (9.0-5.7)		
**IR + YM155**	17.6 ± 6.5	11.9 (17.6-5.7)	8.6 (11.9-3.3)	3.75 (8.6/2.3)

^a^Absolute growth delay, the doubling tumor time of treatment group minus that of control group; ^b^Normalized growth delay, the time of absolute growth delay of tumor in IR combined with YM155 group minus that of YM155 group.

Immunohistochemical analysis demonstrated that YM155 significantly inhibited survivin expression in either IR treated or untreated tumors (Figure 7C, upper panel). The mean density of survivin staining in tumors from YM155 treated groups (YM155: 0.021 ± 0.0087; IR plus YM155: 0.008 ± 0.0036) was significantly lower than that in YM155 untreated groups (Control, 0.061 ± 0.0025; IR: 0.050 ± 0.0044) (*P* < 0.01). The TUNEL assay was performed to quantify apoptosis in tumor sections from different groups. As shown in Figure 7C (middle panel), the mean apoptotic index was 2.8% in the control group, 30.6% in the YM155 group, and 22.2% in the IR group; while the combined treatment group had an apoptotic index of 61.2%, significantly higher than either treatment alone (*P* < 0.01). Furthermore, we performed immunohistochemical staining for cleaved caspase-3. As shown in Figure 7C (lower panel), 57.1% cells were positive for cleaved caspase-3 staining in combined treatment group, compared to 31.2% in YM155 alone group and 10.8% in IR alone group. These data indicated that YM155 radiosensitized ESCC *in vivo*.

## Discussion

In the present study, we have shown that survivin inhibition by YM155 enhanced radiation-induced inhibition of growth of esophageal cancer cells and xenografts. Radiosensitization by YM155 in ESCC is associated with the abrogation of radiation-induced G_2_ checkpoint as well as the attenuation of homologous-recombination-mediated DNA damage repair.

Iwasa *et al.* indicated that radiosensitization of NSCLC cells by YM155 was associated with increased activity of caspase-3, suggesting that YM155 sensitized tumor cells to radiation partly by enhancing radiation-induced apoptosis [23]. Our findings now show that abrogation of G_2_ checkpoint in response to radiation is an additional mechanism of sensitization by survivin inhibitor in esophageal cancer cells. We found that YM155 at nanomolar concentration was sufficient to abrogate G_2_ arrest. It is known that shortening of the G_2_ checkpoint leads to decreased repair of radiation-induced damage that could take place before cell division [24],[25]. In support of this possibility, we found that YM155 significantly increased persistent γ-H2AX expression and suppressed RAD51 recruitment in the nuclei of irradiated ESCC cells. The impairment in DSB repair could enhance the apoptosis induced by irradiation. Consistently, we demonstrated that YM155 enhanced the apoptosis and promoted the cleavage of caspase-3 and PARP in irradiated ESCC cells.

For the radiosensitization of cancer cells, pharmacological agents with the ability to inhibit specific checkpoint components, particularly at the G_2_/M transition, have gained interest recently. UCN-01, AZD7762 and SB-218078 have demonstrated synergy with ionizing radiation to inhibit cancer growth by abrogating G_2_/M checkpoint through selective inhibition of Chk1 [26]–[28]. Caffeine and pentoxifylline were radiosensitizers that disrupted radiation-induced G_2_/M checkpoint by inhibiting the activation of ATM and ATR [9],[11]. DNA damage induced G_1_/S arrest and subsequent Non-homologous end joining (NHEJ) repair are p53-dependent and, thus, are deficient in many tumor cells [29]. Wang *et al.* showed that UCN-01 was much less active in abrogating γ-ray-induced G_2_ checkpoint in MCF-7 cells that had normal p53 function, but it preferentially increased the cytotoxicity of cisplatin in MCF-7 cells defective for p53 function [28]. In addition, Chk1 inhibition preferentially sensitizes HCT116 p53−/− cells (versus HCT p53+/+) to gemcitabine and radiation [30],[31]. Therefore, the cells harboring mutations in p53 are deficient in G1 checkpoint and depend on p53-independent G2 checkpoint for DNA damage repair, rendering them more sensitive to G2 checkpoint abrogation [32]. Similarly, in this study we found that radiation caused typical G_2_/M arrest in both ESCC cell lines, indicating aberrant function of p53 in these cell lines. However, further studies are needed on more types of cells with identified p53 status to evaluate the influence of p53 function on YM155 mediated abrogation of G_2_ checkpoint and enhancement of radiation cytotoxicity.

Although this is the first study demonstrating G2 checkpoint abrogation by a survivin suppressant during radiosensitization, some efforts have previously been made to investigate the contribution of survivin inhibition to cell cycle transition. Transfection of siRNA directed against survivin caused specific G0/G1 phase arrest in hepatocellular and lung cancer cells [33],[34]. However, in gastric cancer, survivin-specific siRNA caused cells accumulation in the G2/M phase and diminished the number of cells in the G0/G1 phase [35]. Yamanaka et al. reported that the concomitant treatment of YM155 relieved docetaxel-induced cell cycle arrest at the G2/M phase and synergistically enhanced the cytotoxic activity of docetaxel [36]. Accordingly, our present findings demonstrate that YM155 abrogates the G2 arrest induced by ionizing radiation, thus resulting in preferential cancer cell death. YM155 probably abrogates G2 checkpoint by inhibiting the Wee1/Mik1 kinases that suppress Cdc2 activation or by activating the Cdc25C phosphatase that, in turn, activates Cdc2 [37].

## Conclusions

Targeting survivin with potent inhibitor YM155 modulates G_2_/M cell cycle checkpoint and mediates radiosensitization of esophageal cancer cells both *in vitro* and *in vivo*. Our preclinical results identify survivin inhibitor YM155 as a novel cell-cycle checkpoint abrogator which provides a rationale for future clinical investigation of therapeutic efficacy of YM155 in combination with DNA-damaging agents. Furthermore, our findings suggest that the crucial role of survivin in cell cycle regulation should be investigated in depth in future studies.

## Methods

### Cell culture and reagents

The human esophageal squamous cell carcinoma cell lines Eca109 and TE13 were obtained from Shanghai Cell Bank (Chinese Academy of Sciences, Shanghai, China). The cells were cultured in RPMI 1640 (Hyclone, Thermo Scientific, MA) supplemented with 10% fetal bovine serum (Hyclone) at 37°C under a humidified atmosphere of 5% CO_2_. YM155 monobromide was purchased from SelleckChem (Houston, TX). For the *in vitro* experiments, YM155 was dissolved in DMSO and diluted in medium to a final DMSO concentration of ≤0.1%. For the *in vivo* experiments, YM155 was dissolved and diluted in saline immediately before administration. Mitotic inhibitor nocodazole was purchased from Beyotime institute of biotechnology (Shanghai, China).

### Irradiation conditions

X-ray radiation was delivered by a 6 MV linear accelerator (Elekta, Stockholm, Sweden) at a dose rate of 250 cGy/min with a source-to-target distance of 100 cm. For tumor irradiation, animals were anesthetized with isoflurane and positioned to place the tumor in the center of 1.0 × 1.0 cm radiation field, with the rest of mice shielded from radiation.

### Cell viability assay

Cells were seeded in 96-well plates overnight, then treated with YM155 at various concentrations (0, 1, 2, 5, 8, 10, 25, 50, 100 nmol/L). Twenty-four and forty-eight hours later, 10 microliters of CCK-8 solution (Cell counting kit-8, Dojindo Molecular Technologies, Gaithersburg, MD, USA) was added to each well. Absorbance was determined at 450 nm after 3 hours of incubation. The viability of cells was calculated as following: Viability = (OD_test group_OD_blank group_)/(OD_control group_OD_blank group_) × 100 %, and IC_50_ (half maximal inhibitory concentration) was calculated from the dose–response curves. All experiments were repeated in triplicate.

### Western blot analysis

Cells were harvested and homogenized in RIPA lysis buffer and centrifuged at 12,000 rpm for 20 min at 4°C. Protein concentrations of the supernatants were determined using BCA Protein Quantification Kit (Vazyme biotech co., Itd.). Western blot analysis was performed as previously described [38], using rabbit polyclonal antibodies to human survivin and c-IAP1 (R&D systems, MN), XIAP and Cyclin B1 (Cell signaling, MA), phosphor-Cdc25C, Cdc2, phospho-Cdc2, cleaved PARP and cleaved caspase-3 (Santa Cruz, CA).

### Clonogenic survival assay

Exponentially growing cells were trypsinized as single-cell suspension and diluted serially to appropriate densities and seeded in triplicate in six-well plates. After cell adhesion, they were treated with 0.1% DMSO (control) or YM155 (5 nM or 10 nM) for 24 h, and then subjected to 0, 2, 4, 6 or 8 Gy X-rays irradiation. The cells were then washed with PBS, cultured in drug-free medium for 14 days, fixed with methanol, and stained with Giemsa. Only colonies containing more than 50 cells were scored. SF (surviving fraction) of each irradiation group was corrected by the PE (plating efficiency) of the nonirradiated control. The cell survival curves were fitted according to a multi-target single-hit model and the survival enhancement ratio (SER) was calculated as the ratio of the mean inactivation dose in control cells divided by the mean inactivation dose in YM155-treated cells. The experiment was repeated for three times.

### Flow cytometry for cell cycle

Eca109 or TE13 cells were harvested after 24 h or 48 h treatment with YM155 (10 nM) and/or 8 Gy X rays, respectively. After washing with ice-cold PBS, the cells were fixed with ice-cold 70% ethanol and stored at −20°C for 1 h. Before analysis by flow cytometry, the cells were washed with PBS, resuspended in a staining solution containing 20 μl RNase A solution and 400 μl propidium iodide staining solution (Vazyme biotech co., Itd). Next, cell cycle distribution assessment was performed using a fluorescence-activated cell sorter (BD FACS Calibur).

### Cdc2-Associated H1 Kinase assay

A total of 4 × 10^7^ cells were lysed in 400 μl lysis buffer (50 mM Hepes/NaOH, pH7.4, 150 mM NaCl, 1% Triton X-100, 1 mM dithiothreitol, protease/phosphatase inhibitor cooktail) on ice for 20 min. The cell lysate was centrifuged at 15,000 rpm for 10 min at 4°C and concentration of protein in the supernatant was determined using a BCA protein quantification kit (Beyotime, Shanghai, China). Cellular extracts were cleared by incubation with protein A or protein G-agarose (Cell Signaling, MA) for 30 min at 4°C. After centrifuging at 2,500 rpm for 5 min at 4°C, the supernatant was incubated with 4 μg Cdc2 antibody (Cell Signaling, MA) and 20 μl protein A/G agarose beads for 2 h at 4°C. The protein-agarose beads and bound immune complexes were then pelleted by centrifugation and immunoprecipitates were washed twice with lysis buffer and twice with wash buffer (50 mM Hepes/NaOH, pH 7.4, 10 mM MgCl_2_, 1 mM dithio-threitol) and then subjected to a Cdc2 kinase assay using histone H1 peptide (Merck Millipore, Darmstadt) as a substrate. The immunocomplex was incubated with 40 μg wash buffer containing 10 μg of histone H1, 10 mM adenosine triphosphate (ATP) for 10 min at 30°C, and then subjected to immunoblotting using phosphoserine/threonine/tyrosine antibody (Abcam, Cambridge, MA).

### Immunofluorescence

Immunfluorescence detection of phospho- H2AX and RAD51 foci was performed to monitor DNA double-strand breaks (DSBs) formation and homologous recombination repair (HRR). Cells cultured on coverslips were treated with 10 nM YM155 and irradiated with a dose of 4 Gy to assure a discrimination of individual nuclear foci in immunofluorescence staining. At indicated time points, the cells were fixed by 4% paraformaldehyde for 20 min at room temperature and permeabilized with 0.1% Triton X-100 for 10 min at 4°C. After blocking with Immunol Staining Blocking Buffer (Beyotime, Shanghai, China) for 1 h at room temperature, cells were incubated with antibody for phospho-H2AX (Ser139) (Millipore/Upstate, Temecula, CA) and RAD51 (EMD Millipore, Billerica, MA) at 4°C overnight, followed by staining with Fluorescein (FITC)-conjugated goat anti-mouse IgG (Jackson Immunoresearch, PA) and Rhodamine (TRITC)-conjugated goat anti-rabbit IgG (Jackson Immunoresearch, PA) for 1.5 h at room temperature. Finally, the samples were counterstained with 2 μg/ml DAPI and mounted in 3 μl of mounting medium (Beyotime). Three random fields each containing 50 cells were examined at a magnification of ×100 under a Zeiss LSM5 confocal laser-scanning microscope (Carl Zeiss, Jena, Germany). Nuclei containing ≥10 immunoreactive foci were scored as positive for γ-H2AX, and ≥5 foci as positive for RAD51.

### Apoptosis analysis

Annexin V-FITC/PI dual staining was performed by using apoptosis detection kit from Keygen Biotech (Nanjing, China) according to the manufacturer’s instruction. Flow-cytometric analysis for induction of apoptosis was performed as previously described [38].

### Xenograft tumor radiosensitivity studies

Animal experiments were approved by Ethics Committee of Nanjing Medical University. Five to six week-old male BALB/C nude mice were provided by Nanjing Medical University Animal Center. A suspension of 1 × 10^6^ Eca109 cells in 0.1 mL PBS was injected s.c. into one site of the right leg of nude mice. Tumors were allowed to grow for 7 days before treatment. Twenty-four nude mice with established tumors (all ~250 mm^3^) were divided into four groups and treated with (a) vehicle (PBS) alone; (b) a single dose of 8 Gy IR; (c) YM155 alone (5 mg/kg as a 7-day continuous infusion); or (d) YM155 plus IR (a single fraction of 8 Gy IR delivered on day 3 of drug treatment). Body weight and tumor diameter were measured three times per week, and tumor volume was determined according to the formula: (length[L] × width[W]^2^)/2. The efficacy of each treatment was evaluated by the volume change during the treatment period. Growth delay time (GD) was calculated as the time for treated tumors to reach double in volume minus the time for control tumors to reach double in volume. The enhancement factor (EF) was then determined as follows: EF = (GD_IR + YM155_–GD_YM155_)/GD_IR_. The first day of treatment was designated as day 0, and observation continued until the day 20. At the end of observation, mice were sacrificed and tumors were fixed in 10% formalin, embedded in paraffin, and cut into 5 μm-thick sections for immnohistochemistry (IHC) and terminal deoxynucleotidyltransferase (dUTP) nick-end labeling (TUNEL) assay.

### Immunohistochemistry

Paraffin-embedded tumor tissue sections were deparaffinized in xylene, rehydrated in graded ethanol, and rinsed twice with PBS. Endogenous peroxidase activity was blocked by incubating sections with 3% hydrogen peroxide in the dark for 15 min. The sections were then incubated overnight at 4°C with polyclonal antibody to survivin (1:50; Abcam, Ltd., Cambridge, United Kingdom) or cleaved caspase-3 (1:100; Cell Signaling, Beverly, MA, USA). After washes, slides were incubated with horseradish peroxidase (HRP)-conjugated anti-rabbit secondary antibody for 1 h at room temperature. Finally, the slides were visualized by incubation with 3, 3′-diaminobenzidine (DAB) (Dako, Hamburg, Germany) and counterstained with hematoxylin (37%). The sections were analyzed under a Zeiss Axiovert A1 light microscope. The intensity for survivin immunoreactitity in tumor cells was evaluated using Image-Pro Plus 6.0 software. Mean intensity was calculated by dividing the integral optical density (IOD) by area. The cleaved caspase-3 labeling index was calculated by dividing the number of activated caspase-3-positive cells by the total number of nuclei (per 1000 tumor cells from four microscopic visual fields).

### TUNEL assay

Paraffin-embedded tumor tissue were deparaffinized, rehydrated and blocked in 3% hydrogen peroxide as above. The slides were permeabilized with TritonX-100 in 0.1% sodium citrate for 10 min. TUNEL staining was performed using the *in situ* cell death detection Kit-POD (Roche Molecular Biology, Mannheim, Germany) according to the manufacture’s protocol. Peroxidase activity was visualized using the liquid DAB substrate chromogen system (Dako), followed by a haematoxylin counterstaining. The percentage of apoptosis was calculated by dividing the number of TUNEL-positive cells by the total number of nuclei (per 1000 tumor cells from four microscopic visual fields).

### Statistical analysis

All data were expressed as the mean ± standard deviation (SD). Statistical differences between groups were determined by the unpaired Student’s *t* test or one-factor ANOVA. All statistical analysis was performed using SPSS statistics 17.0 software (SPSS, Inc., Chicago, IL, USA). A *P* value < 0.05 was considered statistically significant.

## Competing interests

The authors declare that they have no competing interests.

## Authors’ contributions

QQ, CHY, LJ, ZLL, and SXC conceived and designed the experiments; QQ and CHY performed the experiments; QQ, CHY, ZJC, and SXC contributed reagents and wrote the manuscript; CJ, YX, ZC, LJ, XLP, and ZHC provided critical comments on the design of the study, analysis, and of the interpretation of data; all authors supplied data, critically revised, and gave final approval of the article.
